# Does the entorhinal cortex use the Fourier transform?

**DOI:** 10.3389/fncom.2013.00179

**Published:** 2013-12-11

**Authors:** Jeff Orchard, Hao Yang, Xiang Ji

**Affiliations:** ^1^Centre for Theoretical Neuroscience, University of WaterlooWaterloo, ON, Canada; ^2^David R. Cheriton School of Computer Science, University of WaterlooWaterloo, ON, Canada

**Keywords:** entorhinal cortex, fourier transform, path integration, oscillators, neural engineering framework, phase precession, grid cells, place cells

## Abstract

Some neurons in the entorhinal cortex (EC) fire bursts when the animal occupies locations organized in a hexagonal grid pattern in their spatial environment. Place cells have also been observed, firing bursts only when the animal occupies a particular region of the environment. Both of these types of cells exhibit theta-cycle modulation, firing bursts in the 4–12 Hz range. Grid cells fire bursts of action potentials that precess with respect to the theta cycle, a phenomenon dubbed “theta precession.” Various models have been proposed to explain these phenomena, and how they relate to navigation. Among the most promising are the oscillator interference models. The bank-of-oscillators model proposed by Welday et al. (2011) exhibits all these features. However, their simulations are based on theoretical oscillators, and not implemented entirely with spiking neurons. We extend their work in a number of ways. First, we place the oscillators in a frequency domain and reformulate the model in terms of Fourier theory. Second, this perspective suggests a division of labor for implementing spatial maps: position vs. map layout. The animal's position is encoded in the phases of the oscillators, while the spatial map shape is encoded implicitly in the weights of the connections between the oscillators and the read-out nodes. Third, it reveals that the oscillator phases all need to conform to a linear relationship across the frequency domain. Fourth, we implement a partial model of the EC using spiking leaky integrate-and-fire (LIF) neurons. Fifth, we devise new coupling mechanisms, enlightened by the global phase constraint, and show they are capable of keeping spiking neural oscillators in consistent formation. Our model demonstrates place cells, grid cells, and phase precession. The Fourier model also gives direction for future investigations, such as integrating sensory feedback to combat drift, or explaining why grid cells exist at all.

## 1. Introduction

Some neurons in the entorhinal cortex (EC) exhibit spatial firing patterns (Hafting et al., 2005). These neurons, called “grid cells,” spike preferentially when the animal is at points arranged in a hexagonal grid pattern. Before that, neurons in the hippocampus were found to activate when the animal was in a particular location in the environment (O'Keefe and Dostrovsky, 1971; Muller et al., 1987). These neurons are called “place cells.”

Both types of cells, place cells and grid cells, are modulated by the theta rhythm, a pattern of activity that oscillates at between 4 and 12 Hz. Moreover, the frequency at which grid cells oscillate is influenced by the animal's movement. As the animal moves, the frequencies increase slightly. The amount of increase depends on what direction the animal is moving, and how fast (Sargolini et al., 2006). If the animal moves in a cell's preferred direction, the frequency increases more, whereas in the opposite direction, the frequency increases only marginally. The term “velocity-controlled oscillator,” or VCO, denotes a neuron or population of neurons whose activity oscillates, but at a frequency that is modulated by velocity (Welday et al., 2011).

The result of increased frequencies causes the timing of spike bursts to precess in phase relative to the baseline theta cycle (O'Keefe and Recce, 1993; Tsodyks et al., 1996; Geisler et al., 2007). This phenomenon, first described by O'Keefe and Recce (1993), is called “phase precession.”

Combining the ideas of VCOs and grid cells, researchers proposed that the grid patterns might arise from an interference pattern between VCOs (Burgess et al., 2007; Blair et al., 2008). As the animal moves, these VCOs take on slightly different frequencies, and hence their relative phases change.

Phase is the time integral of frequency, just like position is the time integral of velocity. Hence, if a VCO's frequency is proportional to the animal's velocity, then the VCO's phase is proportional to the animal's position. This is the basis for oscillator interference models.

By combining (adding) two VCOs with different frequencies, the result is a beat interference pattern that generates periods of constructive and destructive interference as their phase difference evolves (Blair et al., 2008). Since phase and position are linked, this interference pattern overlays the animal's spatial environment. Combining three VCOs (that differ in preferred direction by multiples of 60°) tends to create a hexagonal grid interference pattern (Burgess et al., 2007; Krupic et al., 2012).

Despite this progress in understanding grid cells, a satisfactory explanation of the relationship between grid cells and place cells remained unclear. As recently as 2008, researchers had only a handful of ideas of how grid cells might combine to produce place cells (Moser et al., 2008). Some have experimented with combining a random selection of grid cells to produce place-cell like behavior (Fuhs and Touretzky, 2006; Solstad et al., 2006). Others suggested that a sum of grid cells could create place cells, but offered only vague justification (O'Keefe and Burgess, 2005; McNaughton et al., 2006). A more detailed proposal argued that place cells resulted from the Moiré interference patterns between small-scale grid patterns (Blair et al., 2007). However, their method involves intricate resealing of so-called “theta cell” grids, which the authors point out as “potentially a serious limitation” (Blair et al., 2007).

A recent study concluded that distributed encoding using grid cells formed a more efficient representation of spatial location than the same number of place cells (Mathis and Herz, 2012). That work is interesting, but does not discuss the mechanisms underpinning these various cell types. A comprehensive review of the various proposed models can be found in Zilli (2012).

A spiking-neuron based model of path integration used Gaussian surfaces to represent place cells, but encoded these Gaussians by their Fourier coefficients (Conklin and Eliasmith, 2005). This implementation takes advantage of the Fourier Shift Theorem (discussed later), moving the Gaussian pattern of excitation around by applying phase shifts to the Fourier coefficients. However, their model does not address grid cells. Can the Fourier Shift Theorem be used in conjunction with grid cells?

**Figure 1:**
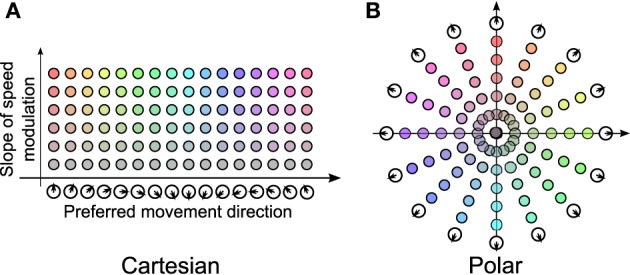
**Cartesian vs. polar representation of VCOs. (A)** The Cartesian arrangement is a derived from part of Figure 7 in Welday et al. (2011). **(B)** The polar arrangement consists of a number of “propellors,” lines of VCOs that pass through the origin. We will refer to the space in **(B)** as the *VCO address space*.

In 2011, Welday et al. showed that “Theta cell burst frequencies varied as the cosine of the rat's movement direction.” In other words, the frequency of each oscillator includes a component proportional to the cosine between the preferred direction vector, and the animal's velocity vector (Welday et al., 2011). They formed a bank of VCOs arranged into a 2-dimensional (2-D) array, where one dimension spans a variety of preferred directions, and the other dimension represents the degree to which frequency is increased by movement. Figure 1A is a recreation of a portion of Figure 7 from their paper.

In their firing-rate model, each VCO is represented as a ring oscillator with a wave of activity that cycles at theta frequency. Hence, each neuron in a ring activates at a particular phase. A read-out node connected to a number of neurons in these ring oscillators will act as a coincidence detector, and fire only when the incoming spikes are sufficiently synchronized. In this sense, a read-out node detects the phase convergence of its inputs.

According to their paper, a read-out node connected to all the phase-matched neurons from rings in the same row could behave like a place cell, and fire only when all the input neurons are in phase with each other. Similarly, choosing only three phase-matched neurons from a row, but with preferred directions separated by 120°, yields a grid cell. Finally, choosing all the phase-matched neurons from rings with the same preferred direction vector can generate a “border cell.”

The authors point out that their model can produce not only grid fields and place fields, but “a sufficiently large number of VCOs should be able to approximate the spatial frequency spectrum of almost any desired spatial function.” However, their justification for this assertion is mathematically unconventional.

Furthermore, they point out that such a system of oscillators is susceptible to drift; before long, these ring oscillators will start to drift out of phase and thereafter fail to produce the desired phase convergence. A coupling mechanism is needed to keep the bank of oscillators in a coherent phase state. Progress has been made to incorporate phase-resetting mechanisms into such models (Monaco et al., 2011). However, the mechanism does not improve the stability of path integration, but rather resets the phases to a previously known state as the animal approaches a landmark.

While the authors hint at the use of Fourier theory, they do not take full advantage of the theory and its interpretations. For example, their arrangement of the VCOs into a rectangular array obscures phase patterns that are revealed by a more conventional frequency-domain layout. They also miss the intuitive link between this frequency domain and hexagonal grid orientation and frequency, and the link between “border cells” and the Fourier Projection-Slice Theorem (Natterer and Wübbeling, 2001).

In this paper, we extend their work in a number of critical ways:

- We express the oscillator states and their interrelations in terms of Fourier theory by arranging the VCOs in a frequency domain.
- Our formulation exposes a linear constraint on the phase relationships between the VCOs.
- We suggest the division of two different components involved in forming activation maps: position is encoded in VCO phases, and map layout is encoded in connection weights.
- We propose and implement novel coupling mechanisms that stabilizes VCO phase relationships.
- We implement a partial model using spiking leaky integrate-and-fire neurons.

## 2. Materials and methods

### 2.1. Fourier model

In this section, we describe how Fourier theory leads to a deeper understanding of the interactions between oscillators. For a primer on the relevant theory, see Appendix A..

The bank-of-oscillators model (Welday et al., 2011) states that a VCO's frequency depends on two parameters: the speed of the animal, and the cosine of the animal's velocity vector with the VCO's preferred direction. In Welday et al. (2011), these VCOs were organized into a 2-D space in which one axis enumerates preferred directions, and the other axis represents the influence of speed (labeled “slope of speed modulation” in Figure 1A).

Another, perhaps more intuitive way of presenting the same 2-D parameter space is to use polar coordinates, as shown in Figure 1B. In this view, each VCO's preferred direction is indicated by its polar angle, while its speed modulation is indicated by its distance from the origin. We will refer to this polar arrangement as the *VCO address space*, and refer to a VCO's address in this space as **d**, using either Cartesian or polar coordinates.

Instead of thinking of a VCO as a ring of neurons, we will think of a VCO as a population of neurons that represents a 2-D unit vector, which we will call a *phase vector* (For more on how a population of neurons can represent a vector, see Appendix B..). Each VCO's phase vector simply rotates around the unit circle at the VCO's specified frequency.

**Figure 2:**
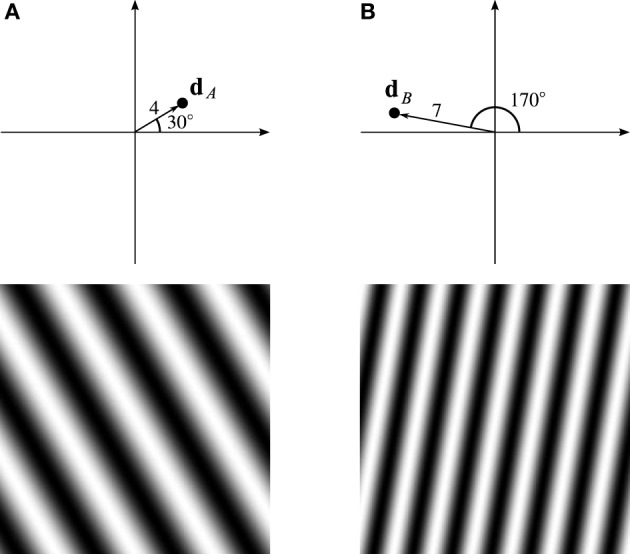
**Trigonometric wave fronts. (A)** Indicates the coefficient at 30°, 4 units from the origin. **(B)** Indicates the coefficient at 170°, 7 units from the origin. The bottom row shows the real part of the Fourier basis function corresponding to the single non-zero Fourier coefficient set to 1.

Consider the VCO with address **d**_*A*_ in Figure 2A, four units from the origin, in the direction of 30°. If the animal's velocity, **v**, is in that direction, the VCO will exhibit a higher frequency than the VCO at the origin. More precisely, the VCO's frequency will increase by an amount proportional to **d**_*A*_ · **v**, the dot-product of **d**_*A*_ and **v**. After a time *t*, the difference in phase between the VCOs at **d**_*A*_ and the origin will be

$$\phi (t)=\int_{0}^{t}d_{A}\cdot \text{v}(\tau )d\tau \;=\;d_{A}\cdot \int_{0}^{t}\text{v}(\tau )d\tau \;=\;d_{A}\cdot x(t).$$

That is, the phase difference depends linearly on the animal's position, **x**(*t*). Even though ϕ is an angle, we will also represent it as a vector on the unit circle. We can write this phase vector as a complex number using Euler's formula,

(cosϕ,sinϕ)≡cosϕ+isinϕ=exp(iϕ),

where $i=\sqrt{-1}$. Thus, we can represent our phase difference as

$$\left(\cos\phi ,\sin\phi \right)=\exp\left(id_{A}\cdot \text{x}\right).$$

Thus, the components of the phase vector trace out sine and cosine wave fronts that are fixed in the animal's environment. The bottom row of Figure 2A shows the wave front corresponding to the VCO located at **d**_*A*_. A different VCO at location **d**_*B*_ traces out a different wave front, as shown in Figure 2B.

**Figure 3:**
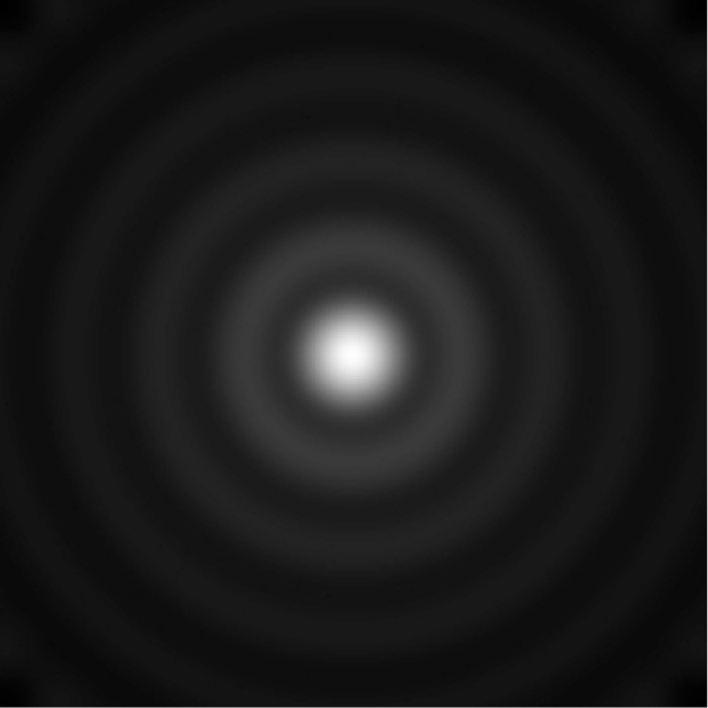
**The spatial function traced out by adding together the whole bank of VCOs**.

Considering that the animal has many such VCOs, what happens if we combine them all into a sum, as if a single read-out node was adding together all the phase vectors from all the VCOs? The value of the read-out node would be

(1) $$\text{p}(\text{x})=\sum_{\rho ,\theta }\exp\left(id_{\rho ,\theta }\cdot \text{x}\right),$$

where **d**_ρ, θ_ is the address of a VCO, and the subscripts ρ and θ index distance from the origin and orientation, respectively. An image created using this simple method is shown in Figure 3. The activity of this read-out node corresponds to a spatial map akin to a place cell. Why is that? The answer has to do with the fact that Equation (1) almost looks like an inverse Discrete Fourier Transform^1^ (Oppenheim and Schafer, 1999). In the following sections, we outline the benefits of thinking about the EC in terms of the Fourier transform, and use it to extend the bank-of-oscillators model.

### 2.2. Entorhinal cortex

Here we outline our model of how the Fourier transform relates to the navigational function of the EC.

We can think of the VCO address space as a 2-D frequency spectrum (frequency domain). In the frequency domain, each location corresponds to a Fourier basis function (wave front), with orientation and frequency dictated by its location, as illustrated in Figure 2. Analogously, each VCO's phase vector traces out a Fourier basis function as the animal moves around, with orientation and frequency dictated by the VCO's address in this space.

A read-out node that adds together the phase vectors of two VCOs receives the sum of those two Fourier basis functions. (Recall that the read-out node is a population of neurons that can encode the resulting vector). Hence, adding these phase vectors together is like adding Fourier basis functions.

**Figure 4:**
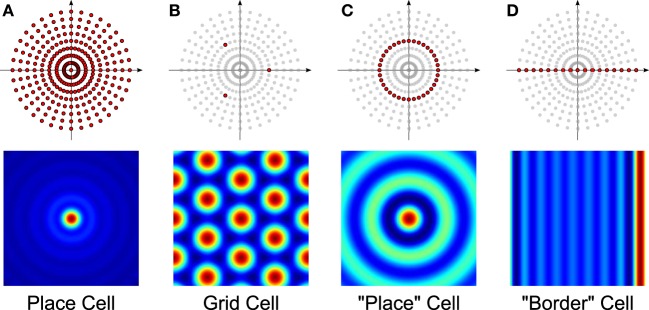
**Sample spatial maps (bottom row) and the selection of VCOs used to generate them (darker dots, top row)**. Each cell type receives input from: **(A)** all VCOs, **(B)** three VCOs arranged in a triad, **(C)** an annulus of VCOs, and **(D)** a line of VCOs.

Different firing-rate spatial maps can be constructed by picking and choosing which VCOs to combine. For example, the bottom row of Figure 4 shows a number of spatial maps similar to those elicited by place cells, grid cells, and “border” cells. The VCOs used to create the maps are colored darker in the top row. For the sake of comparison, we included Figure 4C to show the “place” cell that was proposed in Welday et al. (2011), using only the VCOs from a given spatial frequency (but all orientations).

There is more flexibility though. A read-out node can also combine the VCO phase vectors using different weights, and even apply a phase rotation to individual VCOs. Remember, each VCO and read-out node is a population of neurons that encodes a 2-D phase vector. Using the Neural Engineering Framework (NEF, see Appendix B.), we can scale and rotate a VCO's phase vector by choosing the appropriate connection strengths. Scaling and phase rotation is exactly what a Fourier coefficient does. Hence, combining the VCO phase vectors in this way performs an inverse Fourier transform, where the connection weights are determined by the desired Fourier coefficients.

**Figure 5:**
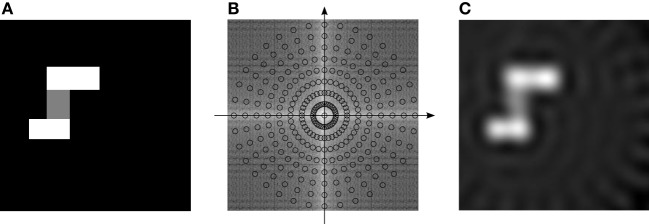
**Example of a general spatial map. (A)** Shows the ideal spatial map. **(B)** Shows the modulus of the spatial map's Fourier coefficients, overlaid with VCO locations (18 propellors, 9 rings). **(C)** Shows the spatial map resulting from combining all the VCOs, each weighted by its corresponding Fourier coefficient.

Consider the following example, illustrated in Figure 5. Part A in the figure shows an idealized spatial activation map, where brightness indicates the desired activity. We wish to use a combination of VCOs (wave fronts) to duplicate—as accurately as possible—that activation map. Figure 5B shows the Fourier transform of the ideal map in A, overlaid with sampling locations that correspond to the VCO addresses. By sampling the Fourier transform at those locations, we get the Fourier coefficients that we should use as weights for the VCO phase vectors. That is, each VCO's phase vector gets transformed by the Fourier coefficient as it is transmitted to the read-out node. Part C of the figure shows the theoretical activity of the read-out node, attained by combining the VCO phase vectors using these Fourier coefficients. Note that a cone filter was also used to compensate for the polar sampling, as is common practice in computed tomography reconstruction (Natterer and Wübbeling, 2001).

Why is this Fourier interpretation helpful? The answer comes from how it decomposes the construction of spatial maps into two parts. The animal's position in its environment is encoded by the phases of the VCOs, while the shape of the spatial map is encoded by the connection weights.

We have already discussed how the connection weights can produce almost any spatial map. Now we will look at how the animal's location is represented by the collective phases of the VCOs.

The Fourier Shift Theorem (described in Appendix A.) states that any function *f* can be shifted by multiplying each of its Fourier coefficients, *F*_*k*_, by a phase ramp, as shown in Equation (A2) in Appendix A. In 2-D, we have two spatial dimensions, (*x, y*), and two frequency dimensions, (*k*, ℓ). The Fourier Shift Theorem states that the Fourier coefficients of our function, shifted by (*x, y*), can be written,

$$G_{\left(k,\ell \right)}=\exp\left(-2\pi i\frac{\left(k,\ell )\cdot (x,y\right)}{N}\right)F_{\left(k,\ell \right)}.$$

**Figure 6:**
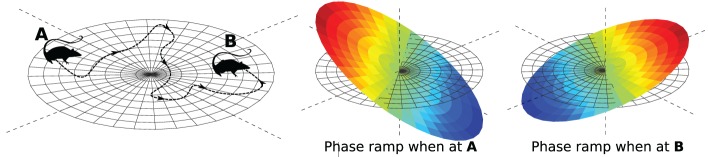
**Encoding location in a phase ramp**. As the animal moves from A to B, the VCOs change their frequencies in such a way that the slope of their phase ramp represents the animal's position (see Equation 2).

Or, more concisely,

(2) $$G_{\text{d}}=\exp\left(-2\pi i\frac{\text{d}\cdot \text{x}}{N}\right)F_{\text{d}},$$

where **d** is the address of the VCO, and **x** is the shift. We shift our function simply by multiplying its Fourier coefficients by a phase ramp. It is called a phase ramp because the expression inside the exponential function is a ramp (or linear function) in 2-D. The slope of the ramp is controlled by **x**. If **x** is the animal's position, then the slope of the ramp changes as the animal moves, as illustrated in Figure 6.

This view of the EC makes clear a strict set of conditions on the VCO phases. They all must change in a way consistent with a linear phase ramp. We can take advantage of this condition. As will be seen, spiking neurons do not make perfect oscillators. In the next section, we will use this constraint to generate coupling mechanisms that stabilize the process.

The results shown thus far are from an idealized implementation of the EC and its bank of VCOs. However, we also implemented a partial version of the EC Fourier model using spiking leaky integrate-and-fire (LIF) neurons (Koch, 1999). Here we describe our implementation of the model, outline the challenges, and display results from simulation experiments.

### 2.3. Dynamics of coupled oscillators

#### 2.3.1. Neural simple harmonic oscillator

**Figure 7:**
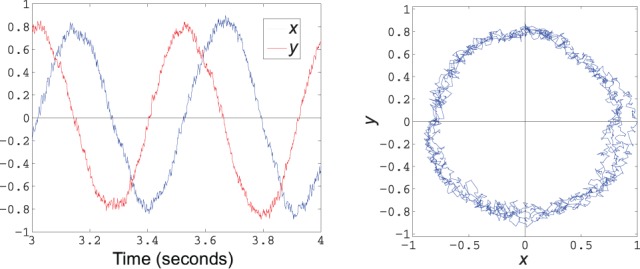
**Decoded state of a simple harmonic oscillator implemented using 200 spiking LIF neurons**. The plot on the left shows the *x* and *y* components over 1 s of time, while the graph on the right plots the phase portrait of *x* vs. *y* over the same time frame.

The simple harmonic oscillator is governed by the system of differential equations

$$\begin{array}{l} \frac{dx}{dt}=cy\\ \frac{dy}{dt}=-cx \end{array}$$

where *c* is a scalar. Solutions to this dynamical system include all circular orbits around the origin in the (*x, y*)-plane. The frequency of oscillation is proportional to *c*. To implement this behavior in neurons, we compute the decoders *D* that decode

$$f(x,y)=[x+\tau_{s}cy,\;y-\tau_{s}cx]$$

(see Appendix B.). The decoded state is immediately fed back into the population, leading to the trajectory shown in Figure 7.

A VCO can be constructed by making the population encode a 3-D vector of the form (*x, y*, θ), where the *x* and *y* components oscillate at frequency (in radians per second) specified by θ. In this case, the decoder would be designed to decode

$$f(x,y,\theta )=\left[x+\tau_{s}\theta y,\;y-\tau_{s}\theta x\right].$$

We used a stabilized version of the simple harmonic oscillator by incorporating a unit-vector constraint into the decoder,

$$f(x,y,\theta )=\frac{\left[x+\tau_{s}\theta y,\;y-\tau_{s}\theta x\right]}{{\left\|\left[x+\tau_{s}\theta y,\;y-\tau_{s}\theta x\right]\right\|}_{2}}.$$

**Figure 8:**
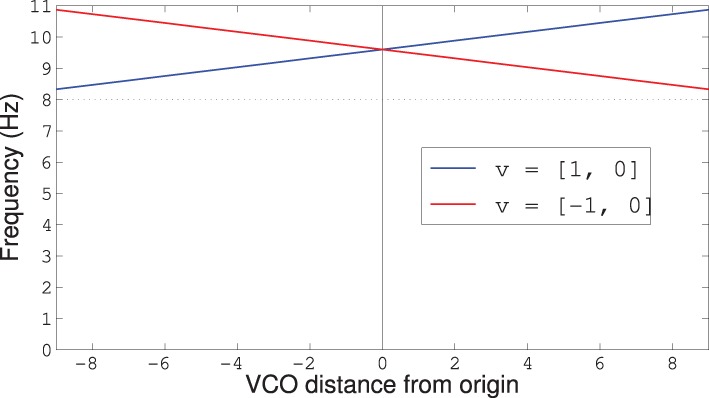
**Frequency modulation for VCOs with preferred direction d_*n*_ = [1,0] and v either [1, 0] or [−1, 0]**. The dotted line shows the baseline theta-frequency of 8 Hz. Notice that all VCOs have a frequency above the baseline 8 Hz, no matter which way the rat is running.

In our model, the VCOs were modeled using populations of 300 LIF neurons. The degree to which the animal's velocity vector influences the frequency of the oscillators depends on where the VCO sits in the plane. Similar to the frequency law stated in Equation (27) of Welday et al. (2011), we derive the frequency of the VCO at location **d**_*n*_ from the animal's velocity vector, **v**, using

(3) $$\theta_{n}=8+1.6\;\|\text{v}{\|}_{2}+1.273\;d_{n}\cdot \text{v}$$

where the vectors **v** and **d**_*n*_ are assumed to be in the unit circle. The coefficient of 1.6 comes from using Equation (27) from Welday et al. (2011) with a maximum speed of 25 cm/s, while the coefficient of 1.273 comes from 4/π, a factor that scales from radians to radius and increases the influence of that term by a factor of 4. Figure 8 shows how the frequencies vary with the VCO's distance from the origin, and that the frequencies are always above the baseline theta-rhythm of 8 Hz no matter which direction the rat is moving.

Our model is composed of 1-D arrays of equally-spaced VCO nodes, each one centered on the origin of the address space, and each one containing 17 VCOs. We will refer to each linear array as a “propellor.” The partial model includes three such propellors.

#### 2.3.2. Phase coupling

The stochastic nature of spiking neurons causes imperfect behavior of the oscillators. If set to the same frequency and started in phase, perfect oscillators will remain in phase. In reality, however, slight errors in frequencies will accumulate and cause the oscillators to drift out of phase. Then, the VCO phases will no longer accurately represent the location of the animal.

Phase drift in VCOs can be corrected, as demonstrated in Monaco et al. (2011). In that work, the animal records a snapshot of the VCO phase offsets for a number of spatial landmarks. Later, as the animal passes near one of the landmarks, the VCOs are smoothly brought into the their corresponding (recalled) phases using error feedback. This approach addresses the incorporation of sensory feedback to correct absolute phase errors, but does not intrinsically stabilize the path integration process.

In this paper, we address the internal consistency of the VCOs, rather than absolute drift. We use phase coupling to maintain an internal state consistent with a phase ramp. Such coupling can be implemented in a number of ways. For brevity, we outline our solution in detail for part of the network (coupling within a single propellor), and include less detail for the other forms of coupling (between propellors).

The absolute phase of the oscillators does not matter. What matters is the phase differences between VCOs. In particular, the phases should follow a linear phase ramp along the propellor, as described by Equation (2). The phases can drift, as long as their relative phases maintain a linear trend across the array.

One way to stabilize the relative phases is to couple the oscillators to each other. We reasoned that the system should be free to allow any linear slope in phase, but discourage other phase differences.

**Figure 9:**
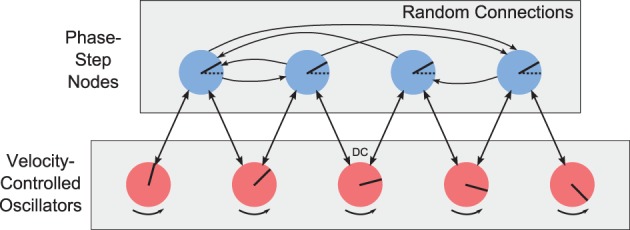
**Array of velocity-controlled oscillators (propellor)**. Each adjacent pair of VCOs is coupled by a phase-step node. The phase-step nodes are randomly connected to each other to arrive at a consensus for what the phase difference should be between adjacent VCOs.

Since the phase should change linearly and the VCO nodes are equally-spaced, then each pair of adjacent VCOs should have the same phase difference, or phase step. For each propellor, we introduce an array of neural populations to couple the oscillators within that propellor. We call these nodes “phase step” nodes. Figure 9 shows the array of VCOs (the propellor), and their connections to an array of phase-step coupling nodes. Each adjacent pair of oscillators is connected to the same phase-step node. Each phase-step node contains 500 LIF neurons, and represents a 6-D vector of the form (*a, b*, α, β, *c, s*), where (*a, b*) and (α, β) are the phase vectors from the two connecting oscillators, and (*c, s*) represents a phase difference of ϕ, where *c* = cosϕ and *s* = sinϕ.

Ideally, all the phase-step nodes for a given propellor should report the same phase difference. In reality, the phase-step array creates a consensus for this phase difference. Each coupling node decodes the phase difference, $\left(\tilde{c},\tilde{s}\right)$, between its afferent VCOs using

$$(\tilde{c},\tilde{s})=(a,b)\cdot \bar{\left(\alpha ,\beta \right)},$$

where (*a, b*) and (α, β) are the oscillator states, and (α, β) is the conjugate of (α, β). If *a* + *ib* = exp(*i*ϕ_*k*_) and α + *i*β = exp(*i*ϕ_*k* + 1_), then their phase difference can be represented by another phase vector,

$$\begin{array}{l} \tilde{c}+i\tilde{s}=\exp\left(i\phi_{k}\right)\exp\left(-i\phi_{k+1}\right),\\ \;=\exp\left(i\left(\phi_{k}-\phi_{k+1}\right)\right). \end{array}$$

Note, however, that the computation is done by the network entirely in Cartesian coordinates, using

$$\begin{array}{l} \tilde{c}+i\tilde{s}=\left(a+ib\right)\cdot \left(\alpha -i\beta \right)\\ \;=\left(a\alpha +b\beta \right)+i\left(-a\beta +b\alpha \right). \end{array}$$

Each phase-step node decodes $\left(\tilde{c},\tilde{s}\right)$ and projects it out to a random subset of other phase-step nodes (including itself). Thus, each phase-step node receives $\left(\tilde{c},\tilde{s}\right)$ from a number of other nodes, each equally weighted with all the weights adding to 1. This weighted-average consensus gets stored in the (*c, s*)-components of each phase-step node's state.

Recall that our phase-step nodes store vectors of the form (*a, b*, α, β, *c, s*). In a perfect world, the VCO states (*a, b*) and (α, β) would differ in phase by exactly (*c, s*). However, there is always some error. To reduce the error, each phase-step node projects phase adjustments back to their connected VCOs. Given the consensus phase difference (*c, s*), we can generate corrected estimates of (*a, b*) and (α, β) using

$$\begin{array}{l} \left(\tilde{a},\tilde{b}\right)\approx \left(\alpha ,\beta \right)\cdot \bar{\left(c,s\right)},\\ \left(\tilde{\alpha },\tilde{\beta }\right)\;\approx \;\left(a,b\right)\cdot \left(c,s\right). \end{array}$$

In other words, we rotate (α, β) clockwise to get (ã, $\tilde{b}$), an approximation of (*a, b*). Likewise, we rotate (*a, b*) counter-clockwise to get $\left(\tilde{\alpha },\tilde{\beta }\right)$, an approximation of (α, β). Then we can compute phase correction vectors,

$$\begin{array}{l} \left(\Delta \alpha ,\Delta \beta \right)=\left(\tilde{\alpha },\tilde{\beta }\right)-\left(\alpha ,\beta \right),\\ \left(\Delta a,\Delta b\right)=\left(\tilde{a},\tilde{b}\right)-\left(a,b\right). \end{array}$$

**Figure 10:**
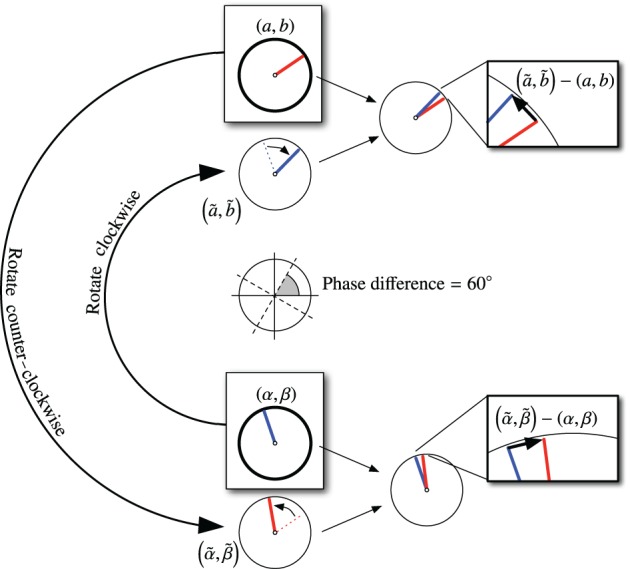
**Calculation for coupling VCOs using phase-step nodes**. In this figure, the consensus phase difference is 60°, as indicated in the center of the figure. The VCO states are shown in the boxes labeled (*a, b*) and (α, β). Each VCO is rotated into alignment (nominally) with the other VCO. The error vectors are fed back as corrections to the VCOs.

This process is illustrated in Figure 10.

Only half of each correction needs to be incorporated to bring the two oscillators into the correct phase relationship. In our implementation, we divide the corrections by 5 and feed them back into the (*x, y*) components of the corresponding VCOs. The divisor of 5 was chosen instead of 2 for stability reasons. In our experience, a wide range of divisors work equally well.

This phase-coupling method maintains a linear progression in phase across each propellor array of VCOs. However, there is nothing keeping the propellors in phase with each other. There are two forms of phase locking required to keep all the propellors consistent.

Notice that all DC nodes should be in phase with each other since there is no direction-dependent frequency modulation on the DC nodes; only the speed affects their frequency. Hence, we need to make sure the phase of the DC components match across all (three) propellors. We achieved this by adding a single 6-D node to perform a coupling similar in nature to the phase-step coupling described above. This DC coupling node acts as a mechanism for finding a consensus phase among the DC nodes. This function could also be accomplished using random connections between DC nodes, similar to how the phase-step nodes arrive at a consensus.

A more complex form of coupling is required to keep the 1-D phase ramps from the individual propellors coplanar with each other. While the phase-step nodes keep the phase linear within a 1-D propellor, we still need a way to ensure that the VCO phases form a linear function (a plane) in 2-D that passes through the origin. For example, drift could cause one propellor to attain a disproportionately steep slope that makes it tilt out of the plane delineated by the other propellors.

In order to ensure that the 1-D phase ramps are coplanar, we chose to couple together three phase-step nodes (from three different propellors). If the propellor orientations are offset by multiples of 60°, the coupling constraint becomes quite simple (omitted here). The resulting phase adjustments are fed back to the phase-step nodes.

### 2.4. Simulation of rat motion

We created our network model to test some specific aspects of the Fourier model. In particular, we wanted to see if we would find grid cells that fired spikes on a hexagonal grid of locations. We also wanted to see if these grid cells would exhibit phase precession compared to a global theta cycle. We added a 2-D VCO node that oscillates at approximately 8 Hz, and used this node's state as the definitive theta cycle.

To simulate the movement of a rat in a circular environment, we added a random-walk function that adjusts the velocity vector smoothly. One could predict the rat's location by numerically integrating the rat's velocity. However, the rat's own perceived location (as encoded in the phase ramp of the EC VCOs) soon drifted away from the computed position. This drift phenomenon has been observed before (Zilli and Hasselmo, 2010) and is probably due to temporal delays in network activity, network transients caused by sudden input changes, and inaccuracies in the frequencies of the VCOs^2^. A real rat seems to avoid this problem by updating its perceived location with sensory information (Burgess et al., 2007). Our model has no sensory input. In terms of assessing the spatial maps of the rat's EC neurons, what is important is where the rat believes it is, not necessarily where the rat actually is Barry et al. (2007) and Blair et al. (2007). We determine the rat's perceived location from the slopes of the phase ramps of the three propellors. In particular, the phase-step nodes encode the slope that we need. Each propellor gives us a projection of the rat's position onto the propellor^3^. Combining the three projections gives us an over-determined system; we find the least-squares solution to get a good estimate of the rat's perceived location.

### 2.5. Network architecture

**Figure 11:**
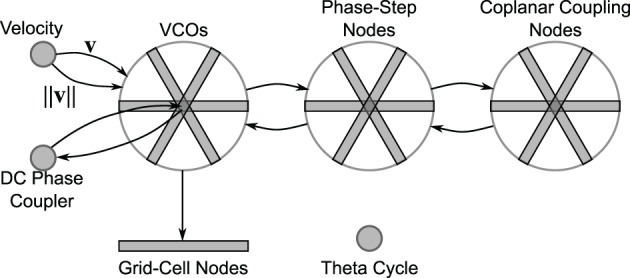
**Network overview**. The velocity node modulates the frequency of the VCOs (see Equation 3). The phase-step nodes couple the VCOs to maintain a 1-D phase ramp within each propellor. The coplanar coupling nodes further keep the phase slopes of the different propellors linearly consistent (so that they all rest in a common plane). The DC phase coupler node keeps the absolute phase of the propellors synchronized. The grid cells sum triads of VCOs. The theta cycle node is a stand-alone oscillator that maintains a frequency of approximately 8 Hz.

As shown in Figure 11, the network consists of three “wheels” of nodes, along with a velocity node, DC phase-coupling node, a theta-cycle node, and an array of grid-cell nodes. Each wheel has three propellors at angles 0°, 120°, and 240° (though a full model would include more propellors per wheel). The first wheel contains 17 VCO populations per propellor. Each population has 300 LIF neurons and encodes a 3-D vector. The recurrent connections of these oscillating populations have a synaptic time constant [τ_*s*_ in Equation (B2) of Appendix B] of 10 ms.

The phase-step wheel also has three propellors, but with 16 nodes per propellor (since they model the phase differences between the VCO nodes). Each phase-step population has 500 LIF neurons and encodes a 6-D vector as described in the previous section. The coplanar coupling wheel mirrors the phase-step wheel, with each coplanar coupling node having 500 LIF neurons and encoding a 6-D vector.

In hopes of observing grid-cell behavior in our model, we created an array of grid-cell nodes (see Figure 11). The grid-cell array contains 17 nodes, mirroring the 17 nodes in each of the VCO propellors. Each grid-cell node (containing 200 LIF neurons) encodes a 2-D vector sum of the phase states from the three corresponding VCOs. That is, each grid-cell node receives the phase state from a triad of VCOs (VCOs at the same radius, but oriented 120° apart) and simply adds them together.

The DC phase-coupler node has 500 LIF neurons and encodes a 6-D vector that duplicates the phases of the three DC nodes. The velocity node has 100 LIF neurons and encodes a 2-D vector. Finally, the theta-cycle node contains 500 LIF neurons and encodes a 2-D vector that oscillates at approximately 8 Hz. The recurrent connections of the theta-cycle population use a synaptic time constant of τ_*s*_ = 5 ms.

Unless otherwise specified, we used the following parameter values for all neurons: synaptic time constant τ_*s*_ = 5 ms, refractory period τ_ref_ = 2 ms, membrane time constant τ_m_ = 20 ms, spiking threshold *J*_*th*_ = 1, encoding vectors (**e**_*n*_ from Equation B1) selected randomly (uniformly) from the unit hyper-sphere, neural gain and bias (α_*n*_ and β_*n*_ from Equation B1) chosen to randomly (uniformly) sample the unit hyper-sphere of the representational space, with a maximum firing rate in the range 200–400 Hz.

## 3. Results

The simulations were run using the Nengo software package (nengo.ca). The whole model includes 119 nodes, for a total of 68,700 LIF neurons. We ran the model for 300 s simulation time. The execution of the model took about 110 min to run on a laptop with a 2.9 GHz Intel Core i7 processor and 8 GB of RAM.

### 3.1. Grid cells

**Figure 12:**
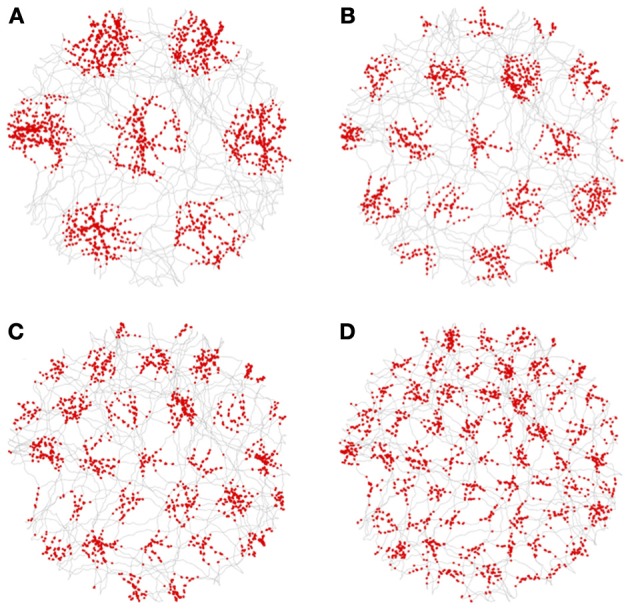
**Spikes (red dots) from grid cells superimposed on the rat's trajectory (gray lines)**. All the grid cells were taken from triads with an orientation of 0°. The neuron in **(A)** is from a grid-cell node at position 2 (where the central, or DC, grid-cell node is index 0). The neurons in **(B–D)** are from grid-cell nodes 3, 4, and 6, respectively.

Figure 12 shows a sampling of activity from neurons in the grid-cell nodes, with their spikes (shown as red dots) superimposed over of the rat's trajectory (shown as gray lines). In the figure, the spatial frequency of the grid-cell triad increases from A to D. Recall that each triad combines the output from the three corresponding VCO nodes, one taken from each propellor. The further from the center (DC), the higher the spatial frequency. The red dots of spikes clearly occur on a hexagonal grid with different scales. Not all neurons in the grid nodes exhibited grid firing patterns. However, about 10% did.

### 3.2. Theta-phase precession

**Figure 13:**
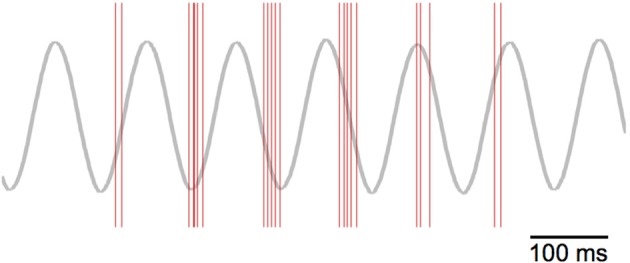
**Theta-phase precession of grid-cell spikes**. The red lines indicate spike events, while the gray line depicts the theta cycle generated by the “theta cycle” node.

If we focus on the timing of the grid-cell spike bursts, we can see that the start of the bursts precess through the theta cycle. Figure 13 plots the spikes as red lines over the theta cycle produced by the “theta” node. The frequency of oscillation for the VCOs—and hence the grid cells—is higher than the nominal 8 Hz theta cycle (as shown in Figure 8). Thus, we see the bursts of grid-cell activity precess through the lower-frequency theta cycle.

## 4. Discussion

The model proposed in this paper extends the model proposed by Welday et al. (2011). We have re-formulated their bank-of-oscillators model using Fourier theory. The VCOs oscillate, so various combinations of them can result in complex interference patterns. Fourier theory is a convenient and powerful tool for understanding these interference patterns.

Our Fourier interpretation points out a global constraint on the VCOs, that they must maintain a phase ramp in the address space. The Fourier Shift Theorem shows us that the location of the animal in its 2-D environment is encoded in the slope of this phase ramp.

Moreover, this phase constraint can be used to generate (or interpret) coupling mechanisms that keep the phases in the proper formation. The VCO phases tend to drift, destroying the location information. Coupling mechanisms that allow phases to change, but only in a manner consistent with a linear phase ramp, maintains the location information.

Each VCO generates a Fourier basis function on the animal's environment. Spatial maps, such as place fields and grid fields, result from the interference patterns between these basis functions. Our Fourier-theory interpretation suggests that the connection weights from the VCOs to the read-out nodes constitute Fourier coefficients. That is, the connections alter the amplitude and phase of each Fourier basis function. Applying a different weight to each VCO is like applying a different Fourier coefficient to each Fourier basis function. Hence, each read-out node performs an inverse Fourier transform.

The inverse Fourier transform implemented by the connections is modulated by the phase state of the VCOs. Since the VCOs are constrained to maintain a linear phase ramp, the spatial maps get shifted (thanks to the Fourier Shift Theorem). The slope of the phase ramp encodes the animal's position in its environment, so the spatial maps are all shifted in concert with the animal's motion. This framework makes it easy to understand the relationship between the VCOs and cells that exhibit spatial maps.

Interestingly, grid cells might be a byproduct of the computation of place cells. All the VCOs in Figure 4A are added together to get the place cell. However, that sum could be done in stages. As an intermediate step to adding all the VCOs together, all possible triads could be added together to form a bank of grid-cell nodes. Then, all those grid cells could be added together to form the place cell. This two-part process is equivalent to adding all of the VCOs together. In addition, variants of grid-like cells have been observed in the EC (Krupic et al., 2012). Just like traditional grid cells, these cells share the property that their spatial activation maps are composed of a small number of Fourier components. That is, their Fourier transforms consist of only a few non-zero Fourier coefficients. Perhaps there is something intrinsically beneficial in combining small sets of Fourier components.

Additionally, grid cells might emerge as a by-product of a phase coupling mechanism. Some research has shown that the distributed nature of grid-cell encoding offers better accuracy than the same number of sparse place cells (Mathis and Herz, 2012). But this theory still does not address *why* grid cells appear, since the bank of VCOs already offers a distributed representation of location. Another theory, and one that we plan to investigate, is that grid cells are a by-product of the coupling mechanisms that maintain the phase relationships within the bank of VCOs. It seems intuitive that place cells could offer a stable and accurate representation of location as long as the underlying network that feeds into the place cells encodes location in a stable and accurate manner. Coupling between nodes harnesses the redundancy in the network and enables wide-spread resources to be focussed on lower-dimensional data, such as location. The coplanar-coupling nodes assess the linear consistency among three or more other nodes. In general, a linearity constraint in 2-D will always require input from at least three VCOs (in addition to the implicitly-included DC node). We plan to investigate more general implementations of the coplanar constraint and observe whether these mechanisms inherently generate grid-cell behaviors.

Couching the behavior of the EC in terms of Fourier theory opens up new vistas of interpretations and predictions. It gives us the mathematical machinery to contemplate other neurophysiological observations. For example, how might sensory feedback be incorporated into the EC? If an animal is given a visual cue of its location, that sensory data might excite the corresponding place cell, which—in turn—could feed back through to the phase-step nodes to adjust the slope of the phase ramp so that it matches the animal's location. It would seem that this feature would be accompanied by a phase-resetting mechanism that allows the VCOs to rapidly realign their phases (or take on some other phase-ramp state). A similar phase-resetting method was able to correct phase drift using error feedback (Monaco et al., 2011). In that work, each landmark has its own phase signature for the VCOs, and as the animal approaches the landmark, its VCOs receive corrective adjustments to bring them into the corresponding phase state. While this approach uses phase adjustments similar to our phase-coupling mechanisms, their method corrects the absolute phase and requires that the entire set of VCO phases be recorded. Their method does not improve the stability of the path-integration process itself. Future work on our Fourier EC model could incorporate these sensory-based error correction mechanisms.

It is well known that different environments elicit different spatial representations in the hippocampus and EC (Barry et al., 2007; Fyhn et al., 2007). While place cells can show entirely different activation maps, grid cells all seem to alter their orientation, scale and phase in concert (Fyhn et al., 2007). Orientation and scale can be accounted for in our model by rotating and scaling the input velocity vector. A phase shift can be implemented by setting the bank of VCOs with an initial phase ramp. As a future investigation, we could consider allowing arbitrary initial VCO phases, similar to the method outlined by Monaco et al. (2011), in which they assign random initial phases to generate independent phase codes for different environments.

The network we have built involves 119 neural populations, and contains a total of 67,800 LIF neurons. Our implementation is an important step in demonstrating the capabilities and behaviors of our model. However, an obvious question remains, how might such a system get established? What self-organizing principles might apply, and where? Spatial maps of place cells have been learned using Hebbian learning (Rolls et al., 2006). Grid cells can emerge spontaneously in a topographically connected network with local excitation and lateral inhibition (Fuhs and Touretzky, 2006; McNaughton et al., 2006). However, these “Turing grids” are not found in adults, leading researchers to suggest that they form during a developmental stage and are used to guide the formation of grid cells in the non-topographical, adult EC network. Even a random selection of grid cells can produce place cells (Solstad et al., 2006; de Almeida et al., 2009). We plan to investigate unsupervised and supervised learning algorithms to derive neural oscillators. We also plan to investigate learning mechanisms that could result in the proper phase-coupling between two or more VCOs.

A recent study found that the dynamics of grid-cell subthreshold membrane potentials not only exhibit theta oscillations, but also systematic depolarizations (so called “ramps”) that coincide with grid fields (Domnisoru et al., 2013). That paper proposes that these depolarizations explain grid fields better than theta interference patterns, posing a challenge to simple oscillator-interference models. The version of our model presented here does not attempt to address those observations; the LIF neuron model we use does not have a facility to change the resting (reset) potential. However, grid cells are not an essential part of our model, and we merely show that they can easily be constructed from the model's raw materials. In the future, it will be interesting to try to incorporate these new observations into an expanded Fourier model of the EC.

## 5. Conclusion

Our Fourier model of the EC path-integration system extends the bank-of-oscillators model proposed by Welday et al. (2011). Using the rich field of Fourier theory, our model suggests the separation of navigation into two components: location, and spatial maps. An animal's location is encoded by the collective phases of the VCOs, which Fourier theory tells us should be arranged in a linear ramp. The slope of the ramp indicates position. The spatial maps that stem from these oscillators can be thought of as inverse Fourier transforms, where the connection weights dictate the corresponding Fourier coefficients. This division of labor allows the EC to maintain a single, dynamic representation of its location, while enabling the construction of many different spatial maps.

The fact that the VCOs all have to maintain a linear ramp in the address space means that this large set of oscillators is actually a distributed encoding of a small piece of data, location. Coupling can keep the VCOs in ramp formation, but allow the slope of the ramp to change freely. We have demonstrated this coupling feature in a system model that uses populations of spiking LIF neurons.

Knowledge about other properties of the Fourier transform can help to guide further development of the model, and assess how it may (or may not) be extended to explain or predict other observations.

### Conflict of interest statement

The authors declare that the research was conducted in the absence of any commercial or financial relationships that could be construed as a potential conflict of interest.

## Figures and Tables

**Figure A1:**
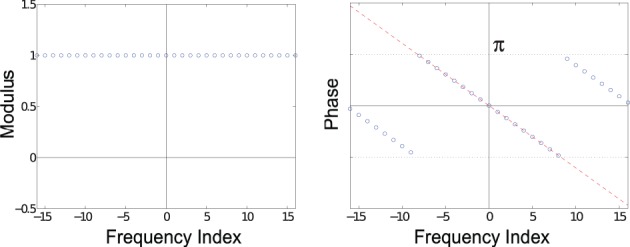
**Fourier transform of δ_2_**. The moduli (left) of the 33 Fourier coefficients are all 1. The phase (right) is linear; the dashed red line shows a linear function, while the blue dots show the same linear function wrapped into the range [−π, π].

**Figure A2:**
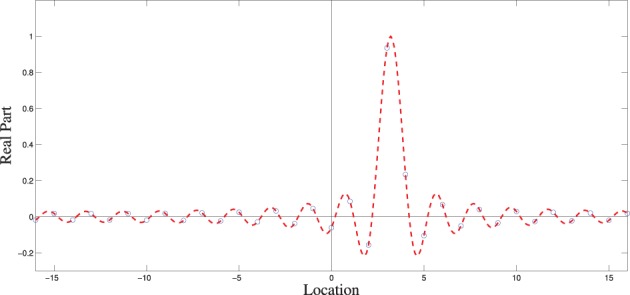
**Fourier interpolation of δ_0_ shifted to the right by 3.2 units**. The dotted red line shows the continuous-domain reconstruction from the phase-shifted Fourier coefficients. The blue circles show the samples produced using the IDFT.

## Footnotes

^1^It would be the IDFT, except that the summation indices correspond to polar coordinates, whereas the IDFT indices use rectangular spacing.

^2^We did not assess the accuracy of the VCO frequencies after implementing the various couplings, though it would be an interesting study.

^3^Proof that the rat's location is projected onto each propellor comes from yet another fascinating and useful property of the Fourier transform, the Fourier Projection Theorem. This property is used heavily in computed tomography reconstruction (Natterer and Wübbeling, 2001).

## Appendix

### A. Fourier theory basics

We will develop the theory using the Discrete Fourier Transform (DFT), but point out that analogous properties exist for the continuous-domain Fourier transform (Oppenheim and Schafer, 1999).

An efficient way to write the Fourier transform is to use complex numbers. Recall that we have two ways of denoting a complex number: using the Cartesian form *a* + *ib*, or the polar form *r* exp(*i*ϕ). In the Cartesian form, we refer to *a* as the *real part*, and *b* as the *imaginary part*. In the polar (or exponental) form, we call *r* the *modulus*, and ϕ the *phase angle*. The polar notation carries with it several advantages. Multiplying *r*_1_ exp(*i*ϕ_1_) and *r*_2_ exp(*i*ϕ_2_) is consistent with the algebraic rules for multiplying exponential functions, giving *r*_1_*r*_2_ exp(*i*(ϕ_1_+ϕ_2_)). In words, you multiply their moduli, and add their phases.

We will start our review of the Fourier transform with a 1-D function. Consider a sampled function *f* with *N* samples indexed *n* = 0, …, *N* − 1,

$$f=\left[f_{0},f_{1},\cdots ,f_{N-2},f_{N-1}\right].$$

The DFT (Oppenheim and Schafer, 1999) of *f* is

$$F_{k}=\sum_{n=0}^{N-1}f_{n}\exp\left(-2\pi i\frac{nk}{N}\right)\;,\;k=0,\ldots ,N-1.$$

Each complex number *F*_*k*_ is called a Fourier coefficient. We can also denote the transform using *F* = DFT(*f*). In essence, the DFT is a frequency decomposition; it takes a spatial signal and represents it as a sum of wave fronts of various frequencies (and orientations, in higher dimensions). Each Fourier coefficient corresponds to a different location in the frequency domain. Each location in the frequency domain represents a different wave front. The value of a Fourier coefficient, *F*_*k*_, represents the contribution of the corresponding wave front. The coefficient *F*_0_ has a special name; it is called the DC, and it always corresponds to the zero frequency and is located at the origin of the frequency domain.

The Fourier basis functions are *N*-periodic. That is,

$$\exp\left(-2\pi i\frac{(n+N)k}{N}\right)=\exp\left(-2\pi i\frac{nk}{N}\right).$$

In other words, it is perfectly valid to refer to *F*_−1_, since *F*_−1_ = *F*_*N* − 1_. Because of this periodicity, we can shift our array of Fourier coefficients and visualize them as being centered on the DC. For example, if *N* is 33, then we list our Fourier coefficients using [*F*_−16_, …, *F*_− 1_, *F*_0_, *F*_1_, …, *F*_16_]. Likewise, use of the DFT implicitly assumes that *f* is also periodic and can be indexed in the same manner. Thus, an equivalent formulation of the DFT is

$$F_{k}=\sum_{n=-\tilde{N}}^{\tilde{N}}f_{n}\exp\left(-2\pi i\frac{nk}{N}\right)\;,\;k=-\tilde{N},\ldots ,\tilde{N}\;,$$

where we assume for simplicity that *N* is odd^4^ and use the symbol *Ñ* to represent $\left\lfloor \frac{N}{2}\right\rfloor$, where the delimiters ⌊·⌋ denote rounding toward zero. We will use this equivalent, centered version of the DFT throughout this paper.

As an example relevant to our purposes, suppose we have a rat that moves along a small corridor, and that we have broken the corridor into *N* blocks, indexed *n* = −*Ñ*, …, *Ñ*. We can represent the location of the rat using an array, δ, indicating where the rat is by placing a 1 in the element corresponding to the rat's location, and setting all the other elements to zero. As the rat moves along the corridor, the array δ changes so that the location of the 1 reflects which block the rat occupies. By this definition, δ is the Kronecker Delta,

$$\delta_{xn}=\{\begin{array}{ll} 1 & \text{if n=x}\\ 0 & \text{if n}\neq \text{x} \end{array}$$

where the subscript *x* represents the index of the block containing the rat, and the subscript *n* indexes the elements of the array δ_*x*_.

The DFT of δ has special properties. The Fourier coefficients of δ_0_ are *F*_*k*_ = 1, all unit-modulus with zero phase. However, when the rat is at position *x*, the DFT of δ_*x*_ is,

$$\begin{array}{l} G_{k}=\sum_{n=-\tilde{N}}^{\tilde{N}}\delta_{xn}\exp\left(-2\pi i\frac{nk}{N}\right)\\ \;=\exp\left(-2\pi i\frac{xk}{N}\right). \end{array}$$

These Fourier coefficients all have a modulus of 1, and their phases vary linearly with *k* (the frequency index). That is, if *G*_*k*_ = *r*_*k*_ exp(*i*ϕ_*k*_), then *r*_*k*_ = 1 and

(A1) $$\phi_{k}=-2\pi \frac{xk}{N}\;.$$

The modulus and phase of δ_2_ are shown in Figure A1. Notice that the phases in Figure A1 form a line (or ramp) as you move along the frequency axis, although the phases are wrapped into the range [−π, π]. The slope of the line is (−2π*x/N*), so the larger *x* is, the steeper the slope. The sign of the slope reflects whether *x* is positive or negative (which direction the rat moved along the corridor).

This property of the DFT is similar to a more general concept known as the Fourier Shift Theorem. The Fourier Shift Theorem tells us how shifting (translating) a signal influences its Fourier coefficients. Suppose that *F*_*k*_ are the Fourier coefficients of a signal *f*_*n*_. Consider a shifted version, *f*_*n − x*_, and its Fourier coefficients, *G*_*k*_. What is the relationship between *G*_*k*_ and *F*_*k*_? The Fourier coefficients of *f*_*n* − *x*_ can be written

$$G_{k}=\sum_{n=-\tilde{N}}^{\tilde{N}}f_{n-x}\exp\left(-2\pi i\frac{nk}{N}\right).$$

Using the change of variables *m* = *n* − *x*, we get

$$G_{k}=\sum_{m=-\tilde{N}-x}^{\tilde{N}-x}f_{m}\exp\left(-2\pi i\frac{(m+x)k}{N}\right).$$

Both exp(·) and *f* are periodic (by assumption, for *f*), so we can shift the summation range without changing its value. Moreover, the exponential function can be split into two components, one containing *m*, while the other can be pulled out of the summation,

(A2) $$\begin{array}{l} G_{k}=\exp\left(-2\pi i\frac{xk}{N}\right)\sum_{m=-\tilde{N}}^{\tilde{N}}f_{m}\exp\left(-2\pi i\frac{mk}{N}\right)\\ G_{k}=\exp\left(-2\pi i\frac{xk}{N}\right)F_{k},\;k=-\tilde{N},\ldots ,\tilde{N}. \end{array}$$

This is the Fourier Shift Theorem, and it tells us that the Fourier coefficients of the shifted signal, *f*_*n* − *x*_, can be derived from the coefficients of the original signal by simply multiplying each coefficient by a phase-shift, where the amount of the phase-shift is a linear function of the frequency index *k*.

Looking back at our example, our rat started at location 0 and the Fourier coefficients of δ_0_ were *F*_*k*_ = 1, *k* = −*Ñ*, …, *Ñ*. However, when the rat moved to location *x*, the Fourier coefficients still had unit modulus, but their phases formed a ramp, exp(−2π*ixk/N*). That is, *G*_*k*_ = exp(−2π*idxk/N*) *F*_*k*_. Applying such a phase ramp can shift any function, not just our δ functions.

The DFT is an invertible transform, and the inverse DFT (IDFT) yields back the original function. For example, IDFT (DFT(δ_*x*_)) = δ_*x*_. With this in mind, we can represent any shifted version of δ_0_ by multiplying its Fourier coefficients by a phase ramp of the desired slope. In other words, the slope of the phase ramp in the Fourier coefficients indicates the shift applied to δ_0_. In this way, the phases of the Fourier coefficients encode the position of the rat.

What if the slope (−2π*x/N*) of the phase ramp along *k* corresponds to a value of *x* that is not an integer? We cannot expect the IDFT to give us δ_*x*_ exactly, since that is only defined for integer values of *x*. As it turns out, non-integer values of *x* yield δ-like signals. The operation is called “Fourier interpolation,” and corresponds to summing the continuous-domain wave fronts and sampling the resulting function. Figure A2 illustrates a non-integer shift, and how the samples computed by the IDFT relate to the underlying continuous-domain Fourier reconstruction.

All of this theory extends trivially to 2-D domains (and higher). A Kronecker delta (or any function, for that matter) on a 2-D domain can be shifted by multiplying its Fourier coefficients by a phase ramp in 2-D.

## B. Neural engineering framework

To build our neural network, we used the Neural Engineering Framework (NEF) (Eliasmith and Anderson, 2003), a powerful and versatile platform that has proven useful for large-scale cognitive modeling (Eliasmith et al., 2012).

The NEF is comprised of three main principles: (1) neural encoding and decoding, (2) neural transformations, and (3) neural dynamics.

### B.1. Neural encoding and decoding

In this framework, data is stored in the collective activities of a population of LIF neurons. The neurons have a range of parameters so that their tuning curves span a wide range of possibilities. A population of *N* neurons (a “node”) can encode a value **x** in its neural activities using

(B1) $$a_{n}(t)=G_{n}\left(\text{x}(t)\cdot {\text{e}}_{n}\alpha_{n}+\beta_{n}\right)$$

where **e**_*n*_ is the encoding vector (preferred direction vector), and α_*n*_ and β_*n*_ are scalar gain and bias terms that account for the neural climate of the neuron. The input to the function *G*_*n*_(·) can be thought of as the input current driving neuron *n*. The function *G*_*n*_ translates the input current to neural activity, either in the form of a firing rate, or a series of spikes. In the case of firing rate, *G*_*n*_ is the steady-state LIF tuning curve,

$$G_{n}(J)=\{\begin{array}{ll} \frac{1}{\tau_{\text{ref}}-\tau_{m}\ln\left(1-\frac{J_{th}}{J}\right)} & \text{for }J>J_{th}\\ 0 & \text{otherwise} \end{array}$$

where τ_ref_ is the refractory period, τ_*m*_ is the membrane time constant, and *J*_*th*_ is the threshold current, below which the neuron has a firing rate of zero. On the other hand, if using spikes, then the output of *G*_*n*_ is represented as a sum of time-shifted Dirac delta functions (Oppenheim and Schafer, 1999),

$$G_{n}(J_{n}(t))=\sum_{p}\delta (t-t_{np}),$$

where *t*_*np*_ is the time of the *p*th spike from neuron *n*. The membrane potential, *v*, is governed by the differential equation,

$$\tau_{m}\frac{dv}{dt}=RJ_{n}(t)-v,$$

where *J*_*n*_(*t*) is the input current, and *R* is the membrane resistance. Once the membrane potential reaches its threshold, the neuron spikes, the timing of the spike is recorded, and the membrane potential is reset to zero.

If we wish to decode the neural activities of a population of neurons, we can compute the optimal linear decoders. We do this by collecting a sampling of inputs, *X*, and corresponding neural activities, *A*. That is, each row of matrix *X* stores a sample input, and each row of matrix *A* stores the corresponding neural activities for all *N* neurons (usually stored as firing rates). To decode from our population, we seek the linear weights *D* that solve

$$\underset{D}{\min}\|AD-X{\|}_{2}^{2}.$$

Thus, the weights in *D* perform a linear transformation from the space of neural activities to the space of input values. This is a linear least-squares problem, and there are multiple ways to compute *D* numerically. Once we have *D*, we can decode neural activities to get an estimate of the value being encoded. Moreover, we can decode arbitrary functions of our encoded data by finding the decoders that solve

$$\underset{D}{\min}\|AD-f(X){\|}_{2}^{2},$$

where *f*(*X*) is a function of the encoded values.

### B.2. Neural transformations

We can use our neural encoders and decoders to transform data from one population *P*, to another population *Q*. To do this, we essentially decode the desired function from *P* and re-encode the result into *Q*. Collapsing those processes together gives the *N* × *M* weight matrix

$$W=D_{P}E_{Q}\alpha_{Q},$$

where *D*_*P*_ is the matrix that decodes the neural activities from the *N* neurons in *P*, and *E*_*Q*_α_*Q*_ is the matrix of *M* encoders for the neurons in *Q*. The weight matrix simply combines the linear decoders and encoders into a single matrix.

### B.3. Neural dynamics

The neural coding and transformation principles above can be built into a dynamic framework by including the temporal action of synapses. When a spike arrives at a synapse, it induces a current on the post-synaptic neuron. We model this post-synaptic current using exponential decay^5^. That is, we convolve an incoming spike train with the post-synaptic filter, *h*(*t*),

(B2) $$h(t)=\frac{1}{\tau_{s}}\exp\left(\frac{-t}{\tau_{s}}\right),$$

where τ_*s*_ is the decay time constant. The total current entering a neuron is a weighted sum over all incoming spike trains so that the total input current arriving at neuron *m* is

$$J_{m}(t)=h(t)⋆\left[\sum_{n}w_{nm}\sum_{p}\delta (t-t_{np})\right],$$

where ⋆ represents convolution, and ω_*nm*_ is the connection weight from neuron *n* to neuron *m*.

We can use a recurrently-connected population of neurons to implement a dynamic model of the form

$$\frac{d\text{x}}{dt}=f(x)$$

by choosing the recurrent connection weights so that they decode and feed back τ_*s*_*f*(**x**) + **x**, where τ_*s*_ is the post-synaptic time constant (Eliasmith and Anderson, 2003).

With these three principles in place, one can implement a dynamical system using spiking LIF neurons by assigning populations to state variables, and connecting them with the appropriate transformations and time constants. A good example of such a dynamical system is the neural simple harmonic oscillator, described in section 2.3.
